# Editorial: alternative antigen processing and presentation in immune disorders

**DOI:** 10.3389/fimmu.2022.993393

**Published:** 2022-09-07

**Authors:** Iñaki Alvarez, Luis C. Antón, Eddie A. James

**Affiliations:** ^1^ Immunology Unit, Department of Cell Biology, Physiology and Immunology, Autonomous University of Barcelona, Bellaterra, Spain; ^2^ Institute of Biotechnology and Biomedicine, Autonomous University of Barcelona, Bellaterra, Spain; ^3^ Centro de Biología Molecular Severo Ochoa, CSIC/UAM, C/Nicolás Cabrera, Madrid, Spain; ^4^ Center for Translational Immunology, Benaroya Research Institute, Seattle, WA, United States

**Keywords:** antigen (Ag), antigen processing and presentation, MHC class I and class II, alternative processing, immune disorders

The key players and processes of the MHC class I and class II pathways were described decades ago and are essential to develop an efficient T cell response. However, they are not confined to canonical pathways only. Indeed, cross-presentation and autophagy are non-canonical processes that allow the presentation of peptides by MHC-I and MHC-II molecules that originate from proteins located in the endocytic or endogenous compartments, respectively. In addition, the structural features of the MHC-peptide interaction were described decades ago. Nevertheless, MHC molecules can bind thousands of different peptides and the complexity of these immunopeptidomes open the door to the study of “non-typical” peptides. Thus, some of them are post-translationally modified peptides, spliced peptides generated by the proteasome, or peptides derived from non-canonical open reading frames. In this special Research Topic issue, we wanted to include an array of manuscripts that provides an extensive view of the alternative processing and presentation by MHC molecules and its involvement in immune disorders. A brief synopsis or each (in topical order) is included below.

Building on their previously reported work, Mansurkhodzhaev et al. investigate the formation of proteasome-generated spliced zwitter peptides originating from self (human) and non-self (viral) antigens. Although such sequences can be envisioned to induce clonal deletion or other tolerance processes and restrain responses against viruses, their analysis suggests that cis-spliced peptides only marginally impinge upon functional anti-viral CD8+ cytotoxic T cell responses.

In his Mini Review Münz highlights basic knowledge about how macroautophagy machinery contributes to antigen processing and presentation, strengthening its noncanonical functions, such as its role in nonconventional phagocytosis and secretion. Therefore, as argued in the manuscript, the multiple tasks played by this machinery makes it a fascinating object of study and, potentially, a powerful target to modulate antigen presentation in pathological situations.

One of the crucial issues in defining the immunopeptidome is the discrimination of signal from noise, which becomes critical when peptide abundance is limiting. Nanaware et al. addressed this issue aiming at the MHC classs II (MHC-II) immunopeptidome in the mouse thymus. They developed a strategy to discard non-binding peptides and to validate bona fide binders, allowing them to identify distinct structural features of thymus MHC-II ligands compared to those from spleen, pointing to differences in antigen processing in these tissues.

Mei et al. presented original research work focused in the need to use computer-based immunoinformatic analysis to predict T cell targets from different strains of SARS-Cov-2 that may be used in future vaccine design. The authors put emphasis in HLA-A*11:01, the most frequent HLA allele in the Chinese population. They experimentally validated three epitopes from Spike protein.

The role that CD8+ T lymphocytes play in immunosurveillance of cancer cells is highlighted by the evasion mechanisms that they develop that affect MHC-I antigen presentation. This is the topic of the exhaustive Review by Dhatchinamoorthy et al.., where they cover different angles from where this issue can be observed: mechanisms of downregulation of MHC-I expression, mutations affecting MHC-I itself or essential components of the antigen processing pathway, the impact of these immunoevasion mechanisms on cancer cell recognition, as well as potential strategies to restore MHC-I expression.

Carré and Mallone elaborate the physiology and function of beta cells and antigen processing and presentation (APP) pathways. Thus, the authors generated some hypotheses on APPs relevant to beta cells, and directed this analysis to T1D pathogenesis. Finally, the authors raised some gaps regarding the HLA-I immunopeptidomes that need to be filled, as their composition in basal or stress conditions or the differences in the antigenic peptides presented by beta cells or dendritic cells during the T cell priming in draining lymph nodes.

The contribution of neuroinflammatory processes to the development of Parkinson’s Disease (PD) has been gaining momentum in recent years. Celastrol, a compound that modulates autophagy and mitophagy, has been proposed to mitigate PD and other neurodegenerative diseases. The work of Ng et al. in this issue explores how this natural product may weaken anti-α-synuclein T cell responses through alterations in the vesicular trafficking of this antigen in dendritic cells. The authors propose that this altered trafficking may preclude its efficient antigen processing, thus thwarting a damaging T cell response.

Ito et al. explore the effects of palladium metal, a well-known allergen, on the surface dynamics of MHC class I protein. Using complementary assays, they demonstrated distinct temporal effects elicited by palladium treatment of murine DC2.4 dendritic cells leading to altered peptide presentation. Inhibition of membrane movement blocked this effect, leading to suppressed Pd-induced T cell-mediated responses.

The impact of sequence defects or variants on the expression and function of MHC molecules provides an important means of understanding the potential impact of natural polymorphisms and acquired mutations. Zhao et al. explore the functional impact of a spontaneous H2-Aa point mutation on MHC II expression and function. They document an atypical type II bare lymphocyte syndrome mediated through a cascade of erroneous mRNA splicing, deletion of eight bases in the mRNA, and a protein frameshift, all leading to a general loss of expression and significantly impaired CD4+ T cell numbers.

In an original research article, Thakur and Luthra-Guptasarma elaborate mechanisms through which some HLA-B27 subtypes are associated with ankylosing spondylitis (AS) and others are not. In their work, they conducted a proteomics-based study to analyze the protein clearance mechanisms in two AS differentially associated alleles (B*27:04, associated, and B*27:09, non-associated). They show that B*27:04 misfolded chains are mainly cleared through activation of the unfolded protein response (UPR), and autophagy. Instead, the B*27:09 misfolded chains are mainly cleared through the endosome-lysosome pathway.

Another topic of great interest is the idea that specific populations of professional antigen presenting cells can play distinct roles in health and disease. Welsh et al. review the often-overlooked role of B cell antigen presentation on the differentiation and longevity of protective memory CD4 T cells. Specifically, they highlight the impact of antigen density and the contributions of MHC Class II accessory molecules (HLA-DM and HLA-DO) to epitope selection and possible impacts on the formation of memory. They propose that low levels of antigen presentation might be to regulate long-term survival of CD4 memory T cells and to prevent cross-reactivity to autoantigens.

In their article, Kang et al. investigate the number and function of Dendritic cells (DCs) in scrub typhus patients. Examining peripheral blood, they observed that plasmacytoid and conventional DCs were numerically deficient and functionally impaired in scrub typhus patients. Alterations in the number and surface phenotype of DCs were absent during disease remission, but phenotypic alterations could be recapitulated through treatment with pro-inflammatory cytokines, suggesting a direct relationship between these alterations and disease severity.

Kawasaki disease (KD) is a severe illness that primarily affects children under the age of five. The condition is characterized by a vasculitis that, if untreated, can cause coronary artery aneurism and adult cardiovascular disease. Intravenous immunoglobulin (IVIG) is an effective treatment for acute KD, but its therapeutic mechanism of action is not completely understood. Wang et al. suggest in their contribution that IVIG treatment reverts the immature (or tolerant) phenotype of circulating myeloid and plasmacytoid dendritic cells, restoring the numbers of peripheral CD4+ T cells, which circulate at reduced levels prior to the treatment.

We would like to dedicate this ResearchTopic to the memory of two pioneers that the Antigen Presentation field lost in the past few years, Enzo Cerundolo and Nilabh Shastri. Their contributions laid the foundations of a knowledge that pervades many concepts used by the papers published in this ResearchTopic.

## Author contributions

EAJ, IA, and LCA each wrote individual sections of the editorial, corresponding to the respective articles that they edited. All three authors organized, revised, and edited the manuscript and agree to its style and content in its final form.

## Funding

IA is supported by Grant RTI2018-097414-B-I00 from the Ministerio de Ciencia e Innovación. LCA is supported by Grant PID2019-110407RB-I00 from the Ministerio de Ciencia e Innovación; the Centro de Biología Molecular Severo Ochoa is funded by institutional grants from Fundación Ramón Areces and Banco de Santander. EAJ is supported by NIH/NIDDK grant no U24 DK104162-08 (Niland) NIAID contract no 75N93019C00068 (Kwok), NIAID grant no 2R01DK081166-11 (Haskins), and an award from the Heidner Foundation.

## Conflict of interest

The authors declare that the research was conducted in the absence of any commercial or financial relationships that could be construed as a potential conflict of interest.

## Publisher’s note

All claims expressed in this article are solely those of the authors and do not necessarily represent those of their affiliated organizations, or those of the publisher, the editors and the reviewers. Any product that may be evaluated in this article, or claim that may be made by its manufacturer, is not guaranteed or endorsed by the publisher.

